# Strangulated Bochdalek hernia: a rare cause of colonic obstruction

**DOI:** 10.1093/jscr/rjag061

**Published:** 2026-02-12

**Authors:** Mohamed Lakhloufi, Yassir Akil, Mohammed Hassani, Anas Derkaoui, Abdelali Guellil, Rachid Jabi, Mohammed Bouziane

**Affiliations:** Department of General Surgery, Mohammed VI University Hospital, PO Box 4806, Oujda University, 60049 Oujda, Morocco; Department of General Surgery, Mohammed VI University Hospital, PO Box 4806, Oujda University, 60049 Oujda, Morocco; Department of General Surgery, Mohammed VI University Hospital, PO Box 4806, Oujda University, 60049 Oujda, Morocco; Department of General Surgery, Mohammed VI University Hospital, PO Box 4806, Oujda University, 60049 Oujda, Morocco; Department of General Surgery, Mohammed VI University Hospital, PO Box 4806, Oujda University, 60049 Oujda, Morocco; Laboratory of Anatomy, Microsurgery and Surgery Experimental and Medical Simulation (LAMCESM), Faculty of Medicine and Pharmacy, Mohammed First University, PO Box 724, Hay Al Quods, Oujda 60000, Morocco; Department of General Surgery, Mohammed VI University Hospital, PO Box 4806, Oujda University, 60049 Oujda, Morocco; Laboratory of Anatomy, Microsurgery and Surgery Experimental and Medical Simulation (LAMCESM), Faculty of Medicine and Pharmacy, Mohammed First University, PO Box 724, Hay Al Quods, Oujda 60000, Morocco; Department of General Surgery, Mohammed VI University Hospital, PO Box 4806, Oujda University, 60049 Oujda, Morocco; Laboratory of Anatomy, Microsurgery and Surgery Experimental and Medical Simulation (LAMCESM), Faculty of Medicine and Pharmacy, Mohammed First University, PO Box 724, Hay Al Quods, Oujda 60000, Morocco

**Keywords:** strangulated Bochdalek hernia, thoraco-abdominal CT scan, complications, surgical emergency

## Abstract

We present the case of a 29-year-old woman who arrived at the emergency department with clinical and radiological evidence of bowel obstruction caused by a strangulated Bochdalek hernia containing colon and epiploic fat. She underwent an emergency exploratory laparotomy. This case highlights a rare cause of colonic obstruction Bochdalek hernia, the diagnosis of which relies primarily on thoracoabdominal computed tomography, and for which prompt surgical intervention is crucial.

## Introduction

Bochdalek hernia is a rare congenital defect resulting from incomplete fusion of the posterolateral diaphragmatic components during embryonic development, most often diagnosed in the neonatal period [1, 2]. In exceptional instances, the defect may remain asymptomatic into adulthood, with diagnosis frequently occurring in the context of acute complications, such as intestinal obstruction secondary to colonic strangulation [3, 4]. Although uncommon, this presentation constitutes a true surgical emergency owing to the high risk of intestinal ischaemia and its potentially fatal sequelae [5, 6].

## Case presentation

**Figure 1:**
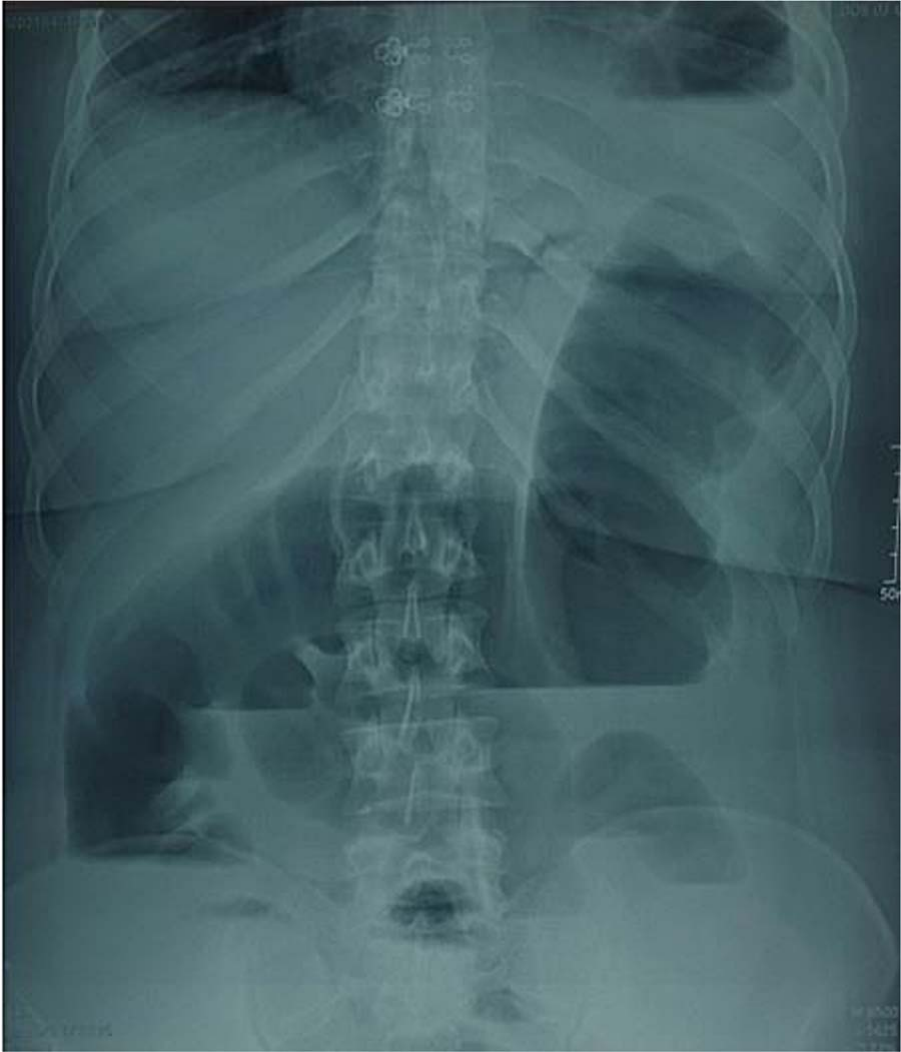
Image of an unprepared abdomen in a standing position, centered on the diaphragmatic domes, showing colonic and small bowel distension with air-fluid levels.

**Figure 2:**
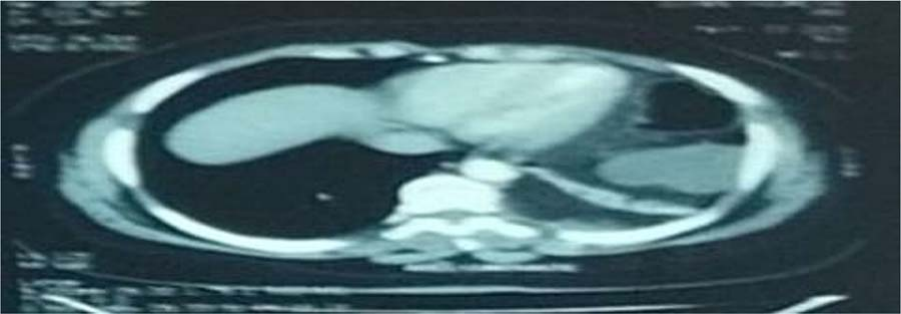
Axial section of an abdominal-pelvic CT scan showing a strangulated Bochdalek hernia with colonic and omental contents.

**Figure 3:**
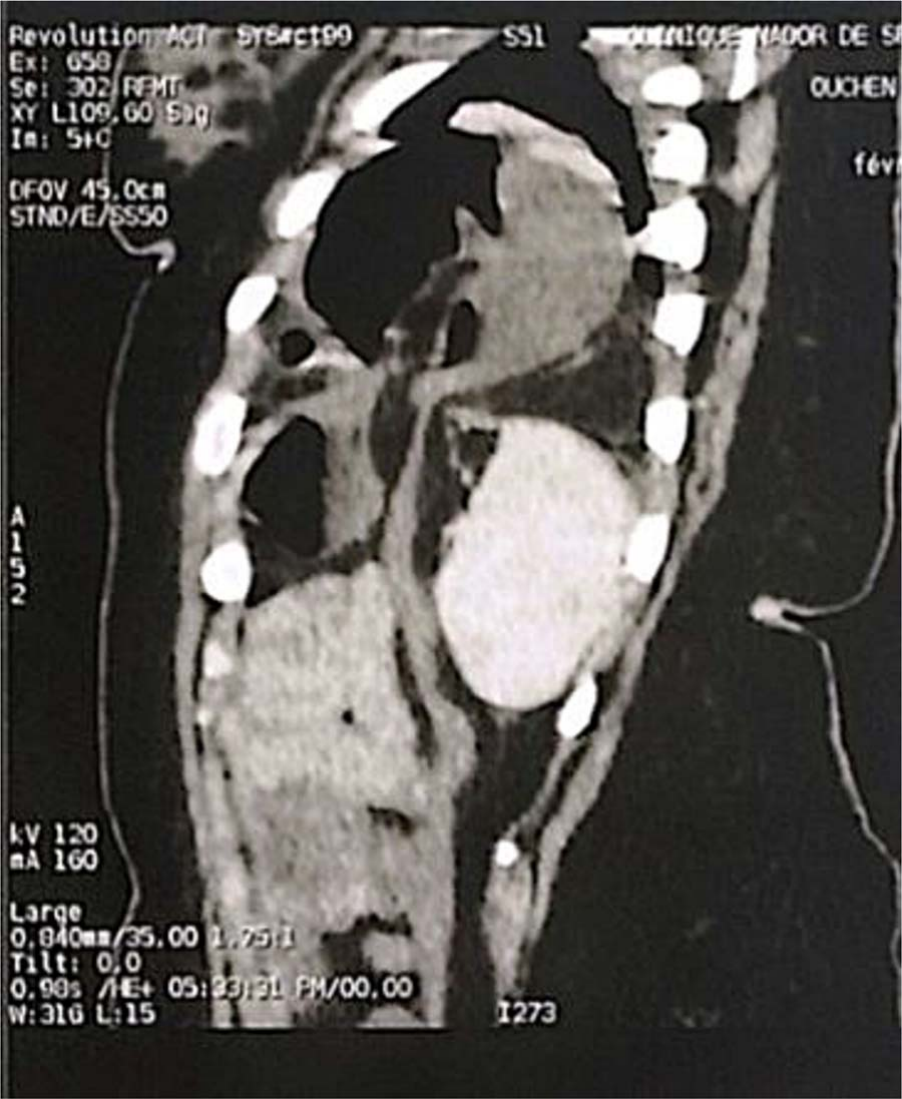
Sagittal section of an abdominal-pelvic angiogram showing a strangulated Bochdalek hernia with colonic and omental contents.

**Figure 4:**
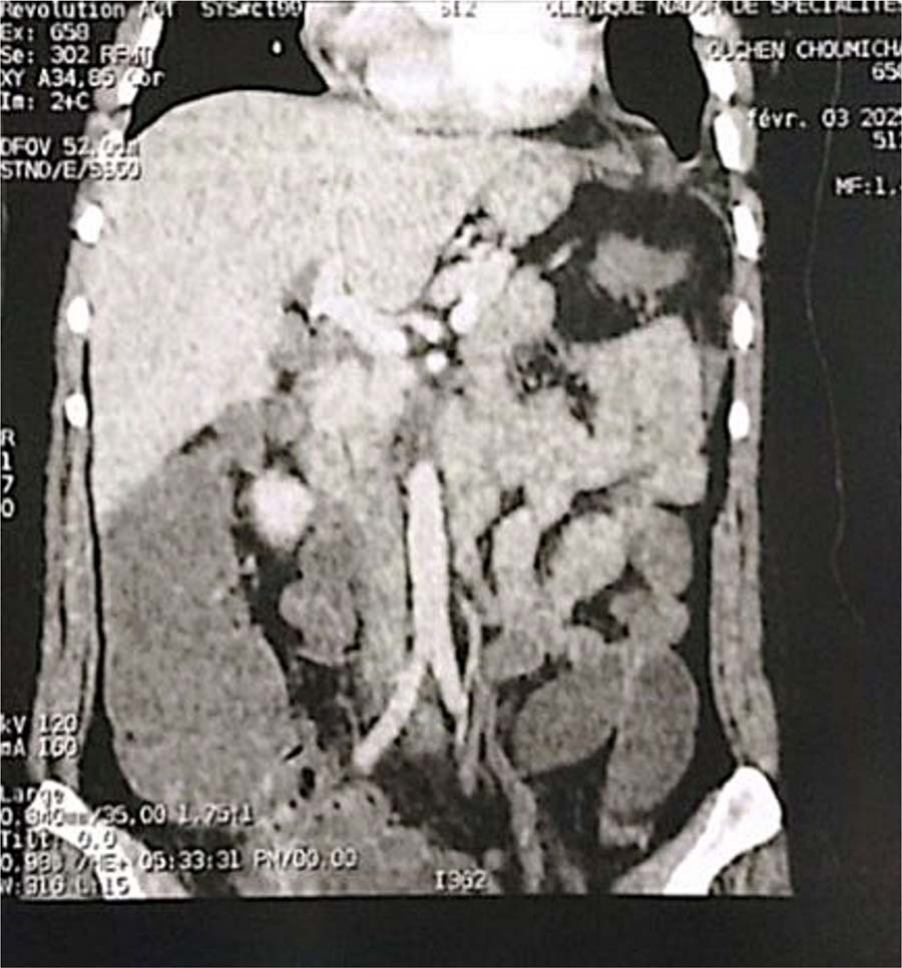
Coronal section of an abdominal-pelvic angiogram showing a strangulated Bochdalek hernia with colonic and omental contents.

**Figure 5:**
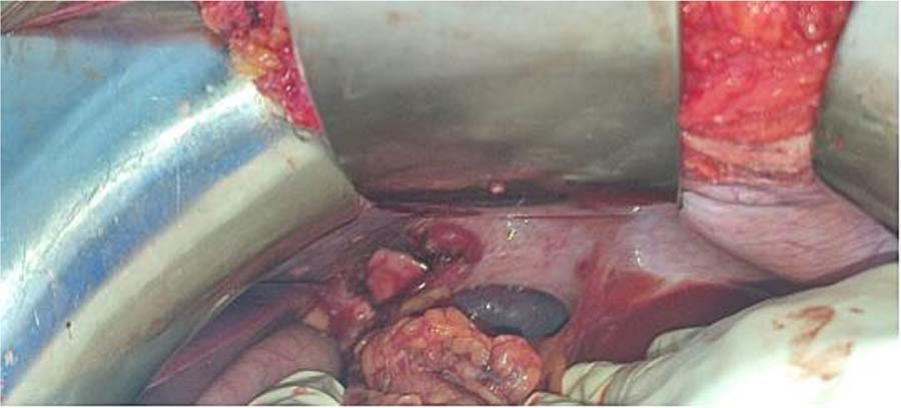
Intraoperative image showing a left Bochdalek hernia.

**Figure 6:**
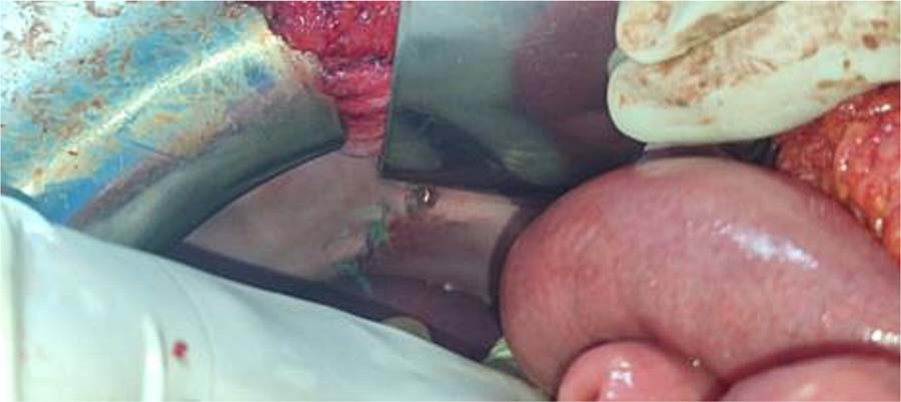
Intraoperative image after suturing of Bochdalek hernia.

A 29-year-old female, with a history of cesarean section performed three months prior and no history of thoracoabdominal trauma, was admitted to the emergency department for an occlusive syndrome characterized by complete cessation of bowel movements and flatus, associated with vomiting, without other digestive or respiratory symptoms. On examination, the patient was conscious, haemodynamically and respiratorily stable. Abdominal examination revealed marked distension with epigastric tenderness, and digital rectal examination demonstrated an empty rectal ampulla. An initial plain abdominal radiograph, performed in the standing position and focused on the diaphragmatic domes, demonstrated colonic and small bowel distension with multiple air–fluid levels (Fig. 1). Subsequently, a thoraco-abdominopelvic CT angiography was performed, revealing a 3 cm left colonic herniation into the thoracic cavity through a partial posterolateral diaphragmatic rupture, associated with moderate left-sided pleural effusion (Figs 2–4). The herniation caused colonic stenosis at the diaphragmatic defect, with upstream distension of small bowel loops, the stomach remaining in its anatomical position (Fig. 5). These findings were consistent with acute intestinal obstruction secondary to a left-sided diaphragmatic hernia. Surgical management consisted of reduction of the herniated contents, assessment of their viability, and closure of the diaphragmatic defect using interrupted nylon sutures (sizes 2/0 and 0) (Fig. 6). Double drainage was instituted: a 28 Fr posteroinferior thoracic drain inserted through a dependent incision in the 5th intercostal space, and a Jackson–Pratt abdominal drain placed in the rectouterine pouch (Douglas’ pouch).

## Discussion

Bochdalek hernia is a congenital diaphragmatic defect resulting from the incomplete closure of the pleuroperitoneal canal. Its occurrence in adulthood is uncommon, with fewer than 200 cases reported in the literature [1, 2]. The condition predominantly involves the left hemidiaphragm (approximately 85% of cases) [1], which is likely attributable to the later closure of this side during embryonic development, as well as the protective effect exerted by the liver on the right side. Bilateral presentations are rare [2].

This condition typically remains asymptomatic for long periods before manifesting with nonspecific digestive symptoms (abdominal pain, vomiting) and respiratory signs (dyspnea, chest pain) [3]. Intermittent presentation is often due to spontaneous hernia reduction. Late-stage complications include intestinal strangulation, as in our case, and the rarer but well-documented gastric volvulus [4, 5], both associated with increased morbidity and mortality rates of up to 32% [6]. Owing to its rarity in adults and nonspecific clinical presentation, imaging is essential for diagnosis.

Standard chest imaging, notably frontal and lateral chest radiographs, typically demonstrates posterolateral basal opacities and may reveal air-fluid levels in cases of digestive herniation. Additionally, displacement of mediastinal structures can be observed, and when the hernia contents are predominantly adipose tissue, a pseudotumoral appearance may be present. However, these radiographic findings on frontal views can be mistaken for pulmonary or pleural neoplasms, while on lateral views, they may mimic a mediastinal mass, emphysematous bulla, pulmonary abscess, or pneumonia. Furthermore, spontaneous reduction of the hernia or the presence of a large defect can simulate a diaphragmatic hernia. Thoracoabdominal CT remains the gold standard for diagnosis, enabling detailed visualization and precise identification of intrathoracic visceral organs, including the small intestine, spleen, liver, kidney, and pancreas. CT imaging also facilitates accurate localization of the posterolateral diaphragmatic defect and, albeit rarely, the detection of a contralateral Bochdalek hernia [2, 3, 7].

The treatment of diaphragmatic hernia is primarily surgical, involving the reduction of herniated contents followed by closure of the diaphragmatic defect using non-absorbable sutures. The selection of the surgical approach is guided by the presence and nature of associated complications. Minimally invasive techniques such as laparoscopy or video-assisted thoracoscopic surgery are recommended in cases without digestive or pulmonary complications. Conversely, laparotomy is preferred when ischemia of the herniated bowel segment is suspected, while thoracotomy is indicated in the presence of respiratory complications or pyothorax to facilitate pleural cavity lavage [7]. Some authors advocate for thoracotomy in right-sided hernias, whereas laparotomy or thoracotomy may be employed for left-sided defects. Postoperative outcomes are generally favorable, with a low incidence of recurrence [7, 8].

## Conclusion

Late-onset Bochdalek hernias are rare congenital defects that may present with diverse clinical symptoms. Among these, digestive complications, as illustrated in our case, are the most commonly documented in the literature. Thoracoabdominal CT scan remains the cornerstone for accurate diagnosis. Surgical repair is the treatment of choice and is essential to prevent further complications.

## Data Availability

The datasets and materials used in this study are available upon request.

## References

[1] Iken M, Mai A, Choukrad F et al. Strangulated bochdalek’s diaphragmatic hernia: a rare cause of acute intestinal obstruction. Pan Afr Med J 2019;34:1–5. 10.11604/pamj.2019.34.90.18427 31934233 PMC6945681

[2] Mullins ME, Stein J, Saini SS et al. Prevalence of incidental Bochdalek’s hernia in a large adult population. Am J Roentgenol 2001;177:363–6. 10.2214/ajr.177.2.1770363 11461863

[3] Habarek M, Belhocine M. Les hernies diaphragmatiques congénitales dites de Bochdalek de révélation tardive: difficultés diagnostiques. J Chir Viscérale 2014;151:A32–3. 10.1016/s1878-786x(14)70182-2

[4] Perhoniemi V, Helminen J, Luosto R . Posterolateral diaphragmatic hernia in adults—acute symptoms, diagnosis and treatment. Case report. *Scand J Thorac Cardiovasc Surg* 1992;26:225–7. 10.3109/14017439209099082 1287838

[5] Habib E, Bellaïche G, Elhadad A. Complications de la hernie de Bochdalek méconnue de l’adulte. Revue de la littérature. Ann Chir 2002;127:208–14. 10.1016/S0003-3944(02)00718-6 11933636

[6] Tschopp J, Jandus P, Purek L *et al.* La hernie de Bochdalek, cause rare de dyspnée et de douleurs abdominales chez l’adulte. *Revue Médicale Suisse* 2009;5:1061–4. 10.53738/REVMED.2009.5.203.1061 19526975

[7] Ramspott JP, Jäger T, Lechner M et al. A systematic review on diagnostics and surgical treatment of adult right-sided Bochdalek hernias and presentation of the current management pathway. Hernia 2022;26:47–59. 10.1007/s10029-021-02445-1 34216313 PMC8881253

[8] Sofi FA, , Ahmed SH, Dar MA et al. Nontraumatic massive right-sided Bochdalek hernia in an adult: an unusual presentation. Am J Emerg Med 2011;29:356.e5–7. 10.1016/j.ajem.2010.03.034 20675093

[9] Harkness MK, Hashim A, Spence D. The ‘hidden’ pneumothorax. Emerg Med J 2004;21:386–7. 10.1136/emj.2003.006080 15107390 PMC1726321

[10] Brown SR, Horton JD, Trivette E et al. Bochdalek hernia in the adult: demographics, presentation, and surgical management. Hernia 2011;15:23–30. 10.1007/s10029-010-0699-3 20614149

